# Neuroprotective Effects of Exercise on Brain Edema and Neurological Movement Disorders Following the Cerebral Ischemia and Reperfusion in Rats

**DOI:** 10.15412/J.BCN.03080110

**Published:** 2017-01

**Authors:** Nabi Shamsaei, Soheila Erfani, Masoud Fereidoni, Ali Shahbazi

**Affiliations:** 1. Department of Physical Education, Faculty of Literature and Humanities, Ilam University, Ilam, Iran.; 2. Department of Biology, Faculty of Sciences, Ferdowsi University of Mashhad, Mashhad, Iran.; 3. Department of Neuroscience, School of Advanced Technologies in Medicine, Iran University of Medical Sciences, Tehran, Iran.

**Keywords:** Exercise, Preconditioning, Edema, Movement disorders, Ischemia and reperfusion

## Abstract

**Introduction:**

Cerebral ischemia and reperfusion causes physiological and biochemical changes in the neuronal cells that will eventually lead to cell damage. Evidence indicates that exercise reduces the ischemia and reperfusion-induced brain damages in animal models of stroke. In the present study, the effect of exercise preconditioning on brain edema and neurological movement disorders following the cerebral ischemia and reperfusion in rats was investigated.

**Methods:**

Twenty-one adult male wistar rats (weighing 260–300 g) were randomly divided into three groups: sham operated, exercise plus ischemia, and ischemia group (7 rats per group). The rats in exercise group were trained to run on a treadmill 5 days a week for 4 weeks. Transient focal cerebral ischemia and reperfusion were induced by middle cerebral artery occlusion (MCAO) for 60 minutes, followed by reperfusion for 23 hours. After 24 hours ischemia, movement disorders were tested by a special neurological examination. Also, cerebral edema was assessed by determining the brain water content.

**Results:**

The results showed that pre-ischemic exercise significantly reduced brain edema (P<0.05). In addition, exercise preconditioning decreased the neurological movement disorders caused by brain ischemia and reperfusion (P<0.05).

**Conclusion:**

Preconditioning by exercise had neuroprotective effects against brain ischemia and reperfusion-induced edema and movement disorders. Thus, it could be considered as a useful strategy for prevention of ischemic injuries, especially in people at risk.

## 1. Introduction

Reduction or cessation of blood flow to a part of the brain and blockage of brain feeding leads to the transient focal cerebral ischemia (Iadecola, Zhang, Xu, Casey, & Ross, 1995). During cerebral ischemia, cerebral blood flow as well as oxygen and metabolite levels reduce, then the reperfusion leads to the return of oxygen to the cells which exhibits superoxide radicals’ generation. It affects the cell signaling and ends in tissue damage (Siesj, 1992).

Tissue damage of a particular organ due to ischemia is exacerbated at the moment of its reoxygenation during reperfusion, a process that is considered to be more harmful than ischemia itself. This mechanism of tissue injury is called reperfusion injury or ischemia-reperfusion (IR) injury (Pinheiro, Holanda, Araujo, & Romaldini, 1999). During reperfusion, the restoration of blood flow is often associated with an exacerbation of tissue injury and an intense inflammatory response (Yellon, Hausenloy, 2007). Reperfusion damage is specifically identified with the development of receptive oxygen species (ROS), endothelial cell damage, expanded vascular penetrability, and the activation of platelets, neutrophils and cytokines (Zimmerman & Granger, 1994).

After the stroke, cerebral edema is one of the important factors responsible for neuronal death and development of the brain lesions (Sharma, Westman, & Nyberg, 1998). Studies indicate that 50% of mortality in severe brain injuries such as stroke or trauma is due the cerebral edema (Bardutzky, & Schwab, 2007). Clinical and experimental studies indicate that development of cerebral edema following the acute focal cerebral ischemia may exacerbate the initial injury (Shaw, Alvord, & Berry, 1959). Cerebral edema may increase intracranial pressure and by pressure on cerebral vessels, reduces blood supply to brain tissue causing the development of lesions (Marmarou, 2004). Also, focal cerebral ischemia in experimental animals is associated with a wide variety of neurological disorders that can affect the animal’s sensory and motor functions (Valen & Vaage, 1993).

Therefore, practical solutions must be found to not only prevent the development of the lesion and cerebral edema, but also reduce the subsequent neurological disorders. Previous studies have shown the beneficial effects of exercise on brain damage caused by ischemia and reperfusion in animal models of cerebral ischemia (Aboutaleb, et al., 2015). However, the protective effects of exercise training from ischemia and reperfusion have still poorly understood. This study aimed to investigate the effects of exercise preconditioning on brain edema and neurological movement disorders following the transient cerebral ischemia in male rats.

## 2. Methods

### 2.1. Animals and experimental groups

Adult male Wistar rats (weighing 260–300g) were (obtained from Tehran Pasteur Institute) housed in standard cages and controlled environment (22°C–24°C, 45%–50% humidity, and 12:12 h light/dark cycle), with free access to food and water. Rats were randomly divided into three groups: sham operation group, exercise plus ischemia group, and ischemia group (7 rats in each group). For induction of ischemia, the animals underwent occlusion of middle cerebral artery (MCAO). In exercise plus ischemia group, the animals spent 4 weeks of exercise before induction of ischemia. Sham operated animals (served as controls) were subjected to the same surgical procedure except for the middle cerebral artery occlusion. All the experiments were performed in accordance with the Helsinki Declaration.

### 2.2. Exercise training protocol

The animals in the physical training group were prepared to keep running on a treadmill (4-path animal treadmill; IITC Life Science Inc., USA) for 4 weeks, 5 days a week. At first, the rats were acclimatized to keep running for 10–15 min at the speed of 5–7 m/min, 0% incline for 2 days before the formal training sessions. The formal treadmill training was begun with a speed of 18 m/min for 35 min and 0° incline for 5 days in first week. The time and intensity of the exercise and treadmill incline were increased progressively, so that the animals were running for 40 min at the speed of 18 m/min with 5° incline, 45 min at the speed of 18 m/min with 10° incline and 50 min at the speed of 18 m/min with 15° incline individually during the second, third and fourth weeks of the exercise training course. At first, electrical stuns (1.0 mA) were expected to constrain the animals to keep running forward. Subsequently, they kept running without electrical incitement. After adaptive running session, the rats began formal training. The rats in the physical training groups were planned to keep running on the treadmill during all 4 weeks. Inactive animals (sham and ischemia groups) were placed on a stationary treadmill daily and were given electrical stimulation in a same way.

### 2.3. Induction of transient focal cerebral ischemia

In this study, we used middle cerebral artery occlusion (MCAO) method for induction of transient focal cerebral ischemia. MCAO was induced by intraluminal filament method in one side (12). Quickly, rats were anesthetized with a blend of ketamine/xylazine (40 mg/kg, IP), and their rectal temperature was kept up at 36.5°C±0.5°C throughout the surgery by utilizing a feedback-directed warming system. Moreover, by applying the intraluminal suture technique, central cerebral ischemia was inducted. A 3-0 nylon suture (Ethicon, Johnsons and Johnsons Intl, Brussels, Belgium), while its tip was adjusted by warming it at almost a fire, was brought into the inner carotid vein through a scratch given in the external carotid artery and progressed 18–20 mm from the common carotid artery bifurcation to hinder the origin of MCA. At that point, after 1 h of MCAO, the intraluminal suture was tenderly removed in order to permit reperfusion. It ought to be said that for 24 h after reperfusion, the animals were kept in individual pens with free access to food and water.

### 2.4. Neurological deficit evaluation

Evaluation of neurological movement deficits was performed 24 hours after MCAO by using a 5-point scoring system, and the following scores were given: normal motor function=0, flexion of contralateral torso or forelimb upon lifting by tail or failure to extend forepaw when suspend vertically=1, circling to the contralateral side but have normal posture at rest=2, loss of righting reflex=3, and no spontaneous motor activity=4 (Khaksari, Aboutaleb, Nasirinezhad, Vakili, & Madjd, 2012).

### 2.5. Brain water content measurement

Brain water content (BWC) was measured as an index of cerebral edema in both ischemic (right) and non-ischemic (left) hemispheres of all the groups. About 24 hours after MCAO, after killing the rats with high doses of ketamine/ xylazine mixture IP injection, the rats’ brains were carefully removed and placed on the pre-cooled rat brain matrix. Then, olfactory bulb, cerebellum, and brain stem were removed. The cerebrum part of the removed brains was divided sagittally into two sections, namely, lesion side and contralateral side. The brains were weighted to obtain wet weight (WW) and then placed in an oven at 110°C for 24 h to determine their dry weight (DW). Brain water content percentage was calculated by using the following formula:

[(WW−DW)/WW]×100

### 2.6. Statistical analysis

All results are reported as Mean±SEM. The Kolmogorov-Smirnov test was used to verify the normality of the variables distribution. One-way analysis of variance (ANOVA) test was used to compare the differences between the groups. When a significant difference was revealed, Scheffe’s post hoc test was used to specify where the difference occurred. The level of significance was set at P<0.05. All data were analyzed by SPSS (SPSS for Windows; Version 16).

## 3. Results

The mortality rate of animals following ischemia and reperfusion was 30% in the ischemia group, 22% in the exercise group, and 0% in the sham group.

### 3.1. The effect of pre-ischemic exercise on ischemia- and reperfusion-induced cerebral edema

**Figure 1:**
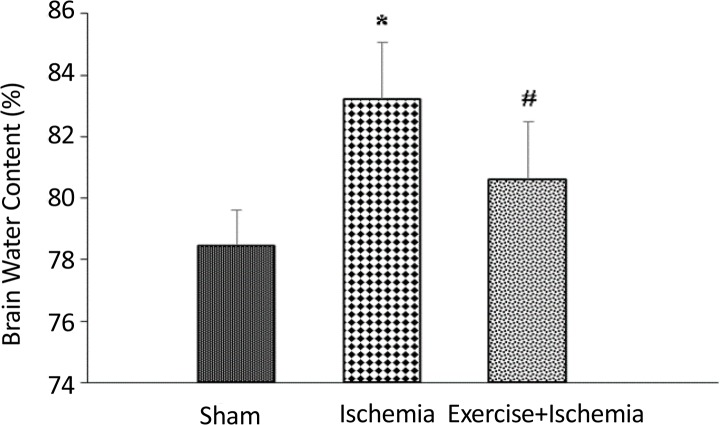
Ischemia leads to an increase in BWC% (edema) while exercise preconditioning attenuates the edema formation. Data were shown as Mean±SEM. *Significant difference compared with the sham group (P<0.01). #Significant difference compared with the ischemia group (P<0.05).

There was no significant difference between the groups with regard to the BWC of the non-ischemic hemisphere of the brain (Table 1). Focal cerebral ischemia and reperfusion induced a significant increase in the BWC of the ischemic hemisphere in the ischemic animals (83.28±1.85%) compared to sham group (78.42±1.14%, P<0.05). This means that cerebral ischemia and reperfusion will cause edema. On the other hand, ischemia- and reperfusion-induced increase in brain water content significantly reduced in ischemic rats preconditioned by exercise (80.57±1.86%) compared to the ischemia group (P<0.05) (Figure 1).

**Table 1.** The brain water content in ischemic and non-ischemic hemispheres in different groups.

**Groups**	**Non-Ischemic Hemisphere**		**Ischemic Hemisphere**	

	**Mean**	**SEM**	**Mean**	**SEM**
Sham	78.42	1.09	78.42	1.14
Ischemia	78.57	1.14	83.28	1.85
Exercise	78.42	1.11	80.57	1.86

### 3.2. The effect of exercise preconditioning on ischemia- and reperfusion-induced neurological movement disorders

**Figure 2:**
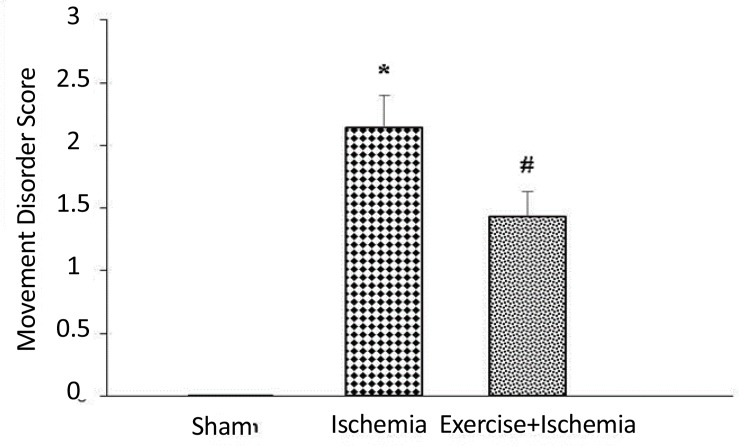
Exercise preconditioning leads to improvement of neurological movement disorders induced by ischemia. Data were shown as Mean±SEM. *Significant difference compared with the sham group (P<0.001). #Significant difference compared with the sham (P<0.001) and ischemia groups (P<0.05).

The results showed that animals in sham group had normal movements and did not show any movement disorder, whereas, 24 hours after ischemia, severe movement disorders were observed in ischemic animals. The mean scores of movement disorders in ischemia group was 2.14±0.26, which indicated the presence of movement disorders due to ischemia. Exercise preconditioning significantly decreased movement disorders scores (1.43±0.2) compared to the ischemia group (P<0.05), (Figure 2).

## 4. Discussion

In this study, we investigated the effects of exercise preconditioning on brain edema and neurological movement disorders following the cerebral ischemia and reperfusion in male rats. The results showed that ischemia and reperfusion increase the brain water content in ischemic hemisphere and cause cerebral edema. Brain edema has an important role in the development of brain lesions (Sharma, et al., 1998) and is one of the most important factors in determining the survival rate of patients in the first hours after stroke. Our findings revealed that preischemic exercise reduced ischemia- and reperfusion-induced cerebral edema. In addition, the findings of this study indicate that cerebral ischemia and reperfusion are associated with neurological movement disorders, and pre-ischemic exercise significantly decreased ischemiaand reperfusion-induced movement disorders.

The underlying mechanism of these neuroprotective effects of physical exercise is still poorly known. Exercise, probably through the amelioration of risk factors, protects brain neurons from ischemia- and reperfusion-induced brain injury. Moreover, exercise can exert endogenous neuroprotection against ischemia and reperfusion injury by preserving neuronal viability (Shamsaei, et al., 2015; Shamsaei, Khaksari, Erfani, Rajabi, & Aboutaleb, 2015).

Studies have indicated that the integrity of the blood-brain barrier has important role in structural resistance and maintaining cerebral vascular permeability. When the blood-brain barrier is damaged by stroke or ischemia, due to changes in the structural proteins (collagen type IV, fibronectin, and laminin), the ability to selectively differentiate the products of cerebrovascular system is impaired and vasogenic edema and changing the properties of neuronal microenvironment can ensue (Del Zoppo, 2010; Del Zoppo & Mabuchi, 2003). Evidently, exercise preconditioning increases the thickness and integrity of the basal lamina and blood-brain barrier (Kochanski, Dornbos, & Ding, 2012). Apparently, one of the factors involved is the collagen type IV. Exercise increases the expression of collagen type IV and reduces neuronal degeneration after stroke in rats (Davis, et al., 2007). Hence, increase in blood-brain barrier resistance can potentially reduce the amount of damage caused by ischemia and reperfusion (Dornbos & Ding, 2012). This action of exercise preconditioning could be considered as a mechanism for exercise effect on edema induced by ischemia and reperfusion observed in the present study.

It is also reported that exercise preconditioning via reducing the matrix metalloproteinases-9 (MMP-9) expression improves the integrity of blood-brain barrier and reduces the damage during ischemia in rats (Chaudhry, et al., 2010). MMP-9 is an enzyme produced by endothelial cells, microglia, and astrocytes. The main role of MMP-9 is degradation of extracellular matrix and basal lamina proteins (Lo, Dalkara, & Moskowitz, 2003). It is unregulated after the onset of ischemia and reperfusion which result in tissue damage, inflammation, and leukocyte infiltration. Moreover, increased expression of MMP-9 leads to cerebral edema by alterations in permeability of the blood-brain barrier (BBB) after cerebral ischemia (Asahi, et al., 2001). Pre-ischemic exercise ameliorates BBB dysfunction and enhances basal lamina integrity in stroke by reducing matrix metalloproteinase (MMP)-9 expression and increasing its endogenous inhibitor (tissue inhibitors of metalloproteinase-1 [TIMP-1]) (Guo, et al., 2008). Based on evidence, heat shock protein (HSP)-70, which its level is increased by exercise preconditioning, is involved in the inhibition of MMPs expression (Shamsaei, et al., 2015). Finally, the long-term exercise preconditioning by reducing the level of MMPs, which are degrading enzyme of the blood-brain barrier, and increasing the expression of collagen type IV and integrins, which are key components of the blood-brain barrier, boosts the integrity of blood-brain barrier and strengthens the neurovascular unit, and ultimately increases the neuronal protection.

The other component of the neurovascular unit affected by exercise preconditioning is brain vessels. Structural changes in brain vessels not only facilitate the delivery of oxygen and essential nutrients to the brain, but also have been shown to reduce brain damage in ischemic injuries (Ding, et al., 2004a). Exercise preconditioning can help improve blood flow during reperfusion after stroke and is associated with a reduction in infarct size (Zwagerman, et al., 2010). Structural changes in the blood vessels take place via angiogenesis and somehow by regulator proteins (such as vascular endothelial growth factor (VEGF), angiopoietin-1 (Ang-1) and angiopoietin-2 (Ang-2). VEGF and Ang1/2 are expressed at high levels after exercise, and these changes will lead to increase in the density of the blood vessels (Ding, et al., 2004a). In this regard, physical exercise increases the level of VEGF protein and mRNA in the hippocampus, but not in the striatum or cerebellum (Tang, Xia, Wagner, & Breen, 2010). Thus, the angiogenesis brought on by exercise preconditioning could induce the neuronal resistance to ischemic injuries by expanding collateral blood flow, thereby enhancing oxygenation and conveyance of dietary and neuroprotective factors to the tissue in reperfusion period (Ding, et al., 2004a).

Inflammation plays a key role in the pathogenesis of cerebral ischemia and reperfusion. Tumor necrosis factor (TNF)-α, as a proinflammatory cytokine, is over-expressed under certain conditions such as stroke and brain injury (Sairanen, Lindsberg, Brenner, Carpen, & Sirén, 2001). TNF-α has been initially considered as a proinflammatory cytokine, but interestingly, in the case of cerebral ischemia, it was found that this cytokine has a neuroprotective effect and is involved in the brain tissue repair (Wang, Yang, & Yu, 2001). Moreover, the role of TNF-α as a potential neuroprotective factor in the animals subjected to exercise preconditioning, is an important issue that should not be ruled out. Evidently, exercise training induces a long-term low rating increment in the concentration of TNF-α, eventually creating neuronal resistance and defense against the ischemia and reperfusion injuries. The underlying mechanisms of this complicated picture of TNF-α have not been discovered completely, but it might include the TNF-α receptors expression. Previous studies have demonstrated that the persistent exercise-induced levels of TNF-α serves to decrease the TNF-α receptor expression after ischemia and reperfusion (Reyes, et al., 2006).

Our suggested neuroprotective effects of exercise, at least in part, conveyed through the upregulation of neurotrophin factors such as nerve growth factor (NGF) and brain derived neurotrophic factor (BDNF). Neural regeneration and networking are examples of the several effects which are attributed to the action of this neurotrophins (Cohen Cory, Kidane, Shirkey, & Marshak, 2010; Kuipers & Bramham, 2006). It has been reported that BDNF and NGF gene expression increases as a result of several weeks of regulated exercise (Ding, et al., 2004b). Overexpression of this neurotrophic factors as a result of chronic exercise training can improve the tolerance capacity of nerve cells opposing cerebral ischemia. Pre-clinical studies in rats showed that exercise could lead to increase in the production of BDNF and NGF in a cerebral ischemia model, and consequently potentiate the resistance of the animal against brain injury induction by cerebral ischemia (Ang, Wong, Moochhala, & Ng, 2003).

In conclusion, the results of this study indicate that pre-ischemic exercise reduces cerebral edema. In addition, exercise preconditioning significantly decreases ischemia- and reperfusion-induced neurological movement disorders. Overall, the results indicate that exercise may exert a neuroprotective effect against ischemia and reperfusion when used as a form of preconditioning. These neuroprotective mechanisms of exercise provide a new therapeutic approach and can be considered as an effective method in reducing ischemia- and reperfusion-induced cerebral complications.
